# *De novo* and quiescent cGVHD are distinguishable in a prognostic biomarker panel

**DOI:** 10.3389/fimmu.2026.1760111

**Published:** 2026-02-20

**Authors:** Lara Vollmer, Katharina M. Habenicht, Andrea Schneider, Matthias Fante, Andreas Mackensen, Julia Winkler, Daniel Wolff, Thomas H. Winkler

**Affiliations:** 1 Department of Internal Medicine 5 – Hematology and Oncology, University Hospital Erlangen, Erlangen, Germany; 2 Department of Biology, Division of Genetics, Nikolaus-Fiebiger-Center for Molecular Medicine, Friedrich-Alexander-University Erlangen-Nuremberg, Erlangen, Germany; 3 Department of Internal Medicine III, University Hospital Regensburg, Regensburg, Germany

**Keywords:** acute GVHD, biomarker, chronic GVHD, disease onset, hematopoietic stem cell transplantation

## Abstract

Chronic graft-versus-host disease (cGVHD) remains the most significant long-term complication after allogeneic hematopoietic stem cell transplantation (allo-HSCT), despite increasing insights into its pathogenesis. The development of reliable prognostic biomarkers is essential for identifying patients at high risk of developing cGVHD which may benefit from pre-emptive intervention. However, valid biomarkers remain elusive, and cGVHD is typically treated after clinical onset only, when irreversible manifestations such as ocular involvement may already be present. In this exploratory study, we identified ten cytokines and chemokines with potential prognostic value for predicting subsequent cGVHD. Using bead-based multiplex analysis, we assessed serum samples from 60 adult allo-HSCT recipients at day +90 and day +180 post-transplant to identify proteins distinguishing patients who later developed cGVHD from those who remained tolerant. Significant differences were found in the serum levels of BAFF, CCL4, CXCL9, and sRAGE between patients with *de novo* cGVHD and those without GVHD. In contrast, elevated IL-6, IL-17A, PAI-1, IL-10, CX3CL1, CXCL1, and CCL4 levels were prognostic for quiescent cGVHD compared with patients with resolved acute GVHD only. These findings underscore the biological heterogeneity of cGVHD and the limited value of single-biomarker approaches, emphasizing the need to consider distinct clinical subgroups and prior disease courses in future predictive models.

## Introduction

Allogeneic hematopoietic stem cell transplantation (allo-HSCT) is a curative therapy for many patients with hematologic malignancies and non-malignant diseases, but continues to be associated with significant mortality (1–3). Chronic graft-versus-host disease (cGVHD) remains the leading cause of long-term morbidity and mortality after allo-HSCT and occurs in 30 – 70% of the transplanted adults or children who survive longer than 100 days (4, 5). Especially, the search for validated biomarkers is of utmost importance (6). In 2014, the National Institute of Health (NIH) updated its biomarker report of 2005 and established a standardized nomenclature for three important groups of biomarkers in cGVHD: 1.) Diagnostic biomarkers that are intended to facilitate the diagnosis of cGVHD, 2.) prognostic biomarkers that can predict disease onset and 3.) predictive biomarkers, used to predict the therapeutic outcome of the patients (7).

In acute GVHD (aGVHD), all three biomarker groups are already well studied, and biomarker panels to facilitate the prognosis and diagnosis of aGVHD are established (8–13). The pathogenesis of chronic disease, by contrast, is much more heterogeneous and can affect nearly every organ, complicating the search for reliable biomarkers (14). Despite advances, the incidence of cGVHD after allo-HSCT remains remarkable, highlighting the urgent need for reliable biomarkers to identify at-risk patients and guide pre-emptive therapies (6, 15). Given that a significant proportion of patients start treatment with already non-reversible manifestations, prognostic biomarkers identifying patients before highly morbid forms of cGVHD manifest and permitting pre-emptive treatment are urgently needed (16). In recent years, several studies have attempted to find diagnostic biomarkers for cGVHD. In this regard, CXCL9, CXCL10, and BAFF have been identified as promising markers; however, these studies have often yielded conflicting results (17–20). In order to classify prognostic biomarkers in cGVHD only a few studies have been conducted. Despite the undeniable clinical potential of this type of biomarker, no reliable single protein has been validated to date (21–25).

Moreover, many biomarker studies have focused on identifying markers without considering the prior history of GVHD. The separation of cGVHD in the two groups: 1.) *de novo* cGVHD (cGVHD without prior acute GVHD) and 2.) quiescent cGVHD (patients with prior acute GVHD and subsequent cGVHD) is not only important regarding the outcome of these patients (26). It may also be relevant in the search for prognostic biomarkers, as different immunological processes might be operating during the development of the disease in these two groups (22, 27).

Therefore, in this study, we focused on identifying potential prognostic biomarkers for cGVHD in patients after allo-HSCT and compared these biomarkers between the two different cGVHD subcohorts: *de novo* cGVHD and quiescent cGVHD. Thereby, we included biomarkers already identified in aGVHD as well as cGVHD and explored their prognostic potential in cGVHD. Moreover, we searched for new, promising candidates that had not been previously described.

## Materials and methods

### Patients

**Figure 1:**
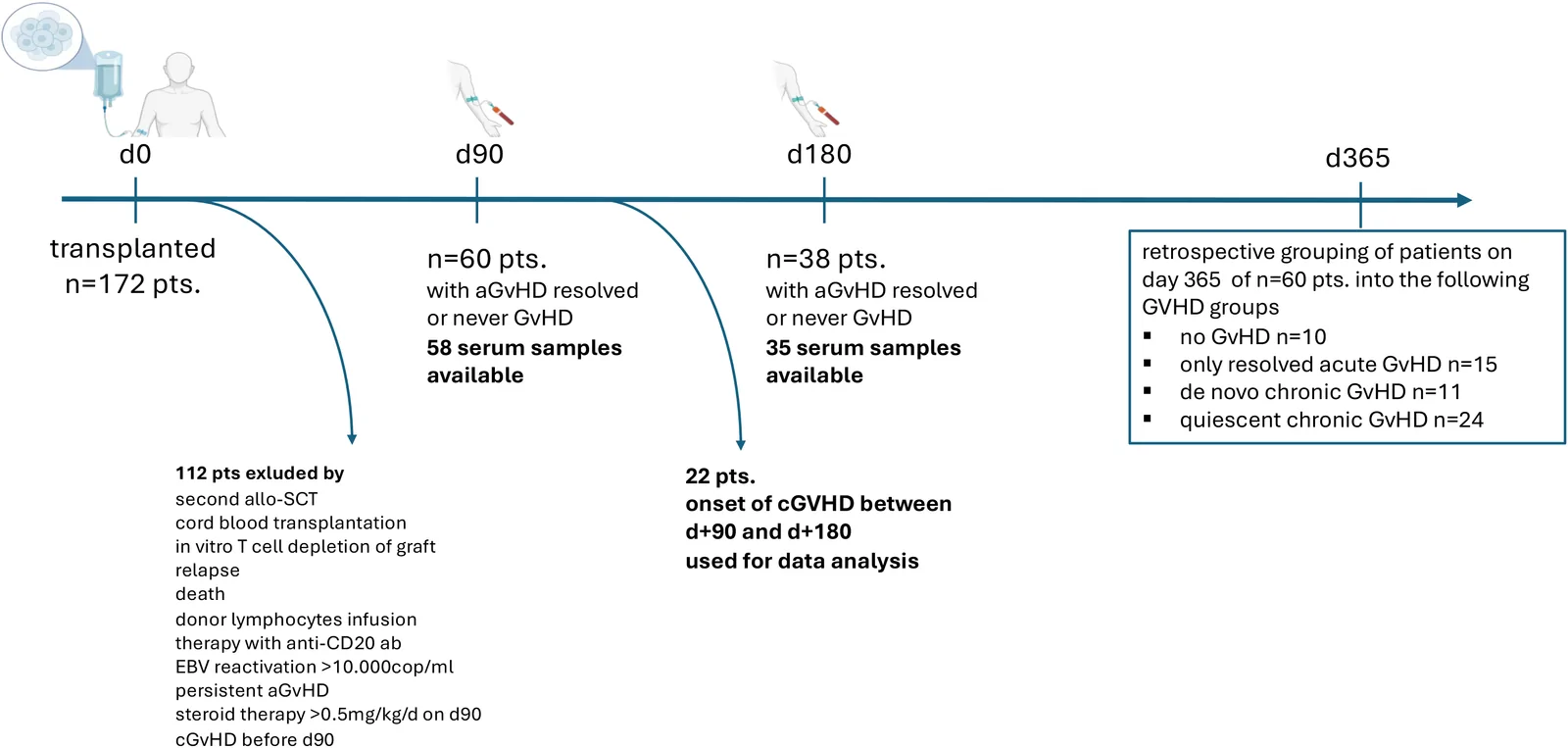
CONSORT diagram and study design. Of 172 allogeneic HSCT recipients prospectively enrolled, 60 were included in the final analysis after applying exclusion criteria (relapse by day +365, donor lymphocyte infusion, early cGVHD before day +90, active aGVHD at sampling, or corticosteroids >0.5 mg/kg/day at sampling). Among these, serum was obtained at day +90 in 58 patients. In 35 patients who did not develop cGVHD between day +90 and day +180, a second sample was collected at day +180.

In a two-center prospective study, 172 allogeneic transplant recipients were enrolled at University Hospital Erlangen (UHE; n=73) and University Hospital Regensburg (UHR; n=99) between November 2017 and March 2020 after informed consent. The study was approved by the local ethics committees (UHE 173-17B; UHR 17-624). Eligible patients had undergone allo-HSCT; exclusions were cord blood transplantation, *in vitro* T-cell–depleted grafts, EBV reactivation >10, 000 copies/mL, or post-transplant rituximab (**Figure 1**). After additional exclusions (relapse within 1 year post-transplant, donor lymphocyte infusion, late-acute or active aGVHD at assessment, or corticosteroids >0.5 mg/kg/day at sampling), 60 patients were included in the final analysis (**Figure 1**). Detailed characteristics are provided in **Table 1**. 27 patients did not receive any corticosteroids at day +90, and the remaining patients received low dose (0.03 – 0.46 mg/kg/day, median 0.19 mg/kg/day).

**Table 1:** Patient characteristics.

Characteristic	All patients (n= 60)	Patients included +90d (n= 58)	Patients included +180d (n= 35)
Age at Tx, yrs			
Median (Range)	53 (19-71)	53 (19-71)	55 (19-69)
Gender, n (%)			
female/male	16 (27%)/44 (73%)	21 (36%)/37 (64%)	12 (34%)/23 (66%)
Donor type, n (%)			
Matched	33 (55%)	33 (57%)	20 (57%)
Mismatched	20 (33%)	19 (33%)	11 (32%)
Haploidentical	7 (12%)	6 (10%)	4 (11%)
Diagnosis, n (%)			
Acute leukemia^a^/MDS^b^	39 (65%)	37 (64%)	25 (71%)
MM^c^/lymphoma^d^	9 (15%)	9 (16%)	4 (12%)
MPD^e^	10 (17%)	10 (17%)	5 (14%)
Non-malignant^f^	2 (3%)	2 (3%)	1 (3%)
Stem-cell source, n (%)			
PBSC^g^/BM^h^	52 (87%)/8 (13%)	52 (90%)/6 (10%)	31 (89%)/4 (11%)
Conditioning regime, n (%)			
myeloablative/reduced	52 (87%)/8 (13%)	50 (86%)/8 (14%)	30 (86%)/5 (14%)
GvHD prophylaxis, n (%)			
CNI^i^+MTX^j^/CNI+MMF^k^	24 (40%)/36 (60%)	24 (41%)/34 (59%)	13 (37%)/22 (63%)
additional ATG^l^/postCy^m^	39 (65%)/16 (27%)	39 (67%)/14 (24%)	23 (66%)/10 (29%)
Acute GvHD, n	39	37	21
Median onset, d (range)	27 (10-77)	28 (10-77)	33 (12-77)
I-II/III-IV, n (%)	35 (90%)/4 (10%)	33 (89%)/4 (11%)	19 (90%)/2 (10%)
Chronic GvHD (*de novo*/quiescent), n	35 (11/24)	34 (11/23)	13 (5/8)
Median onset, d (range)	190 (91-365)	189 (91-365)	255 (195-365)
mild/moderate/severe risk, n (%)	11 (31%)/18 (52%)/6 (17%)	10 (29%)/18 (53%)/6 (18%)	3 (24%)/5 (38%)/5 (38%)
cGVHD treatment, n (%)			
Non or topic steroids	10 (29%)	10 (29%)	4 (31%)
systemic steroids	5 (14%)	5 (15%)	3 (23%)
steroid refractory (other immunosuppressives)	20 (57%)	19 (56%)	6 (46%)

^a^ Includes acute lymphoblastic leukemia and acute myelogenous leukemia.

^b^ Myelodysplastic syndrome.

^c^ Multiple myeloma.

^d^ Includes Hodgkin lymphoma and non-Hodgkin lymphoma.

^e^ Myeloproliferative disorder.

^f^ Includes aplastic anemia and lymphomatoid granulomatosis.

^g^ Peripheral blood stem cells.

^h^ Bone marrow.

^i^ Calcineurin inhibitor.

^j^ Methotrexate.

^k^ Mycophenolate mofetil.

^l^ Anti-thymocyte globulin.

^m^ Post-Cyclophosphamide.

Serum was collected at day +90 (n=58) and day +180 (n=35) post-transplant. At both time points, included patients either had no GVHD or had resolved aGVHD and had not yet developed cGVHD. All samples from patients who ultimately developed cGVHD were obtained before disease onset, ensuring the prognostic nature of the biomarker analyses. 22 patients developed cGVHD before day +180, and serum from 35 patients was analyzed at the second timepoint (see **Figure 1**).

Patients were allocated one year after transplantation in 4 different cohorts, following the classical Glucksberg criteria for aGVHD and the NIH consensus criteria 2014 for cGVHD: 1) never GVHD (n=10), patients who developed neither acute nor chronic GVHD, 2) *de novo* cGVHD (n=11), patients developing cGVHD without prior aGVHD, 3) resolved aGVHD (n=15), patients developing aGVHD but no subsequent cGVHD, and 4) quiescent cGVHD (n=24), patients developing aGVHD and quiescent cGVHD, respectively.

### Sample preparation

Peripheral blood samples were collected using serum blood collection tubes. After an incubation time of at least 30 min to allow the samples to clot, the tubes were centrifuged at 1, 300 rpm for 5 min to separate serum and blood cells and serum was stored at -20 °C until use.

### Detection of cytokines and chemokines

To detect cytokines and chemokines in the serum samples, mix and match Human B cell, Human Proinflammatory Chemokine and Human Inflammation LEGENDplex arrays (Biolegend) were used, following the manufacture´s instructions. All steps were performed according to the manufacturer’s instructions, except a prolonged overnight incubation time after adding the beads to the samples to accommodate for lower protein levels in sera. All parameter analyzed are listed in supp. **Table 1**.

### Statistical analysis

Differences between groups were compared using the unpaired Mann-Whitney-U test, and receiver operating characteristic areas under the curves were estimated nonparametrically using GraphPad Prism (V9.5.1).

## Results

This study aimed to identify prognostic biomarkers for subsequent cGVHD developing after aGVHD or *de novo* without preceding aGVHD. Therefore, patients were separated into four different cohorts: group 1.) neither aGVHD nor cGVHD (label “never GVHD”), group 2.) *de novo* cGVHD without prior aGVHD *(de novo* cGVHD*)*, group 3.) aGVHD but no cGVHD (resolved aGVHD) and group 4.) quiescent cGVHD after previous aGVHD (quiescent cGVHD). Serum samples collected before cGVHD onset enable the identification of biomarkers that predict disease risk, underscoring their prognostic rather than diagnostic value. To ensure biomarkers were not specific to aGVHD, all forms of aGVHD (classic or late) had to be fully resolved at the time of sample collection, and prednisolone doses had to be below 0.5 mg/kg/day due to its known effect on sBAFF levels (18).

### Prognostic biomarkers for the development of cGVHD

**Figure 2:**
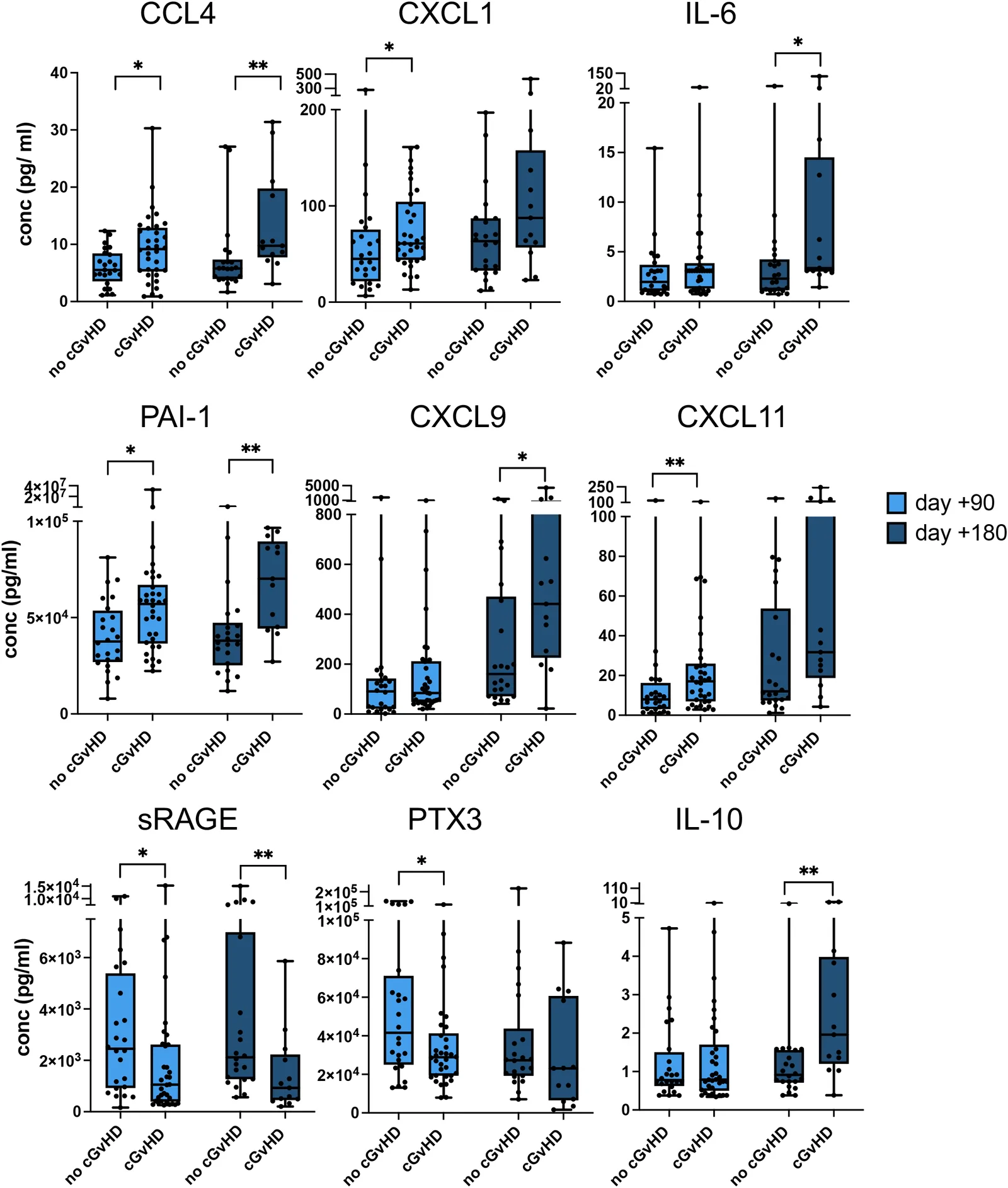
Cytokines and chemokines with prognostic increases or decreases in patients developing cGVHD versus no cGVHD. Serum concentrations of CCL4, CXCL1, IL-6, PAI-1, CXCL9, CXCL11, sRAGE, PTX3, and IL-10 at +90 days (light blue) and +180 days (dark blue) after allo-HSCT are shown as box plots. p-values indicated as *p < 0.05, **p < 0.01, by the Mann-Whitney U test.

First, we analyzed data on prognostic biomarkers for the occurrence of any form of cGVHD versus no cGVHD (aGVHD only or no GVHD at all) at two different time points. In serum samples from 60 patients, we found a total of 9 proteins with significant differences in patients developing cGVHD compared to the controls (**Figure 2**). Among them upregulated were CCL4 and CXCL1 representing chemokines important for the recruitment of macrophages and neutrophils to inflammatory sites, IL-6 as an inflammatory cytokine, and plasminogen activator inhibitor-1 (PAI-1), which is upregulated by proinflammatory stimuli. These pro-inflammatory proteins were significantly higher in the +90d and/or +180d serum samples. Both chemokines CXCL9 and CXCL11, previously shown to be robust biomarkers for newly diagnosed cGVHD were also shown to have some prognostic value, CXCL9 for the +180d timepoint and CXCL11 for the +90d timepoint in our cohort (**Figure 2**). Interestingly, we observed a substantial reduction of soluble receptor for advanced glycation end-products (sRAGE), previously not associated with cGVHD prognosis or diagnosis. Pentraxin-3 (PTX3), which is found elevated in aGVHD, was lower in patients developing cGVHD (28) Finally, IL-10, as well, is a known anti-inflammatory cytokine that was found elevated at the +d180 time point (**Figure 2**).

The strength of association of each significant biomarker was measured by Receiver Operating Characteristic (ROC) analysis based on the Area under the ROC curve (AUC). All of the above-mentioned elevated proteins showed an AUC significantly greater than 0.5 (**Table 2**).

**Table 2:** ROC analysis of the prognostic serum biomarkers and the occurrence of cGVHD versus no GVHD or aGHVD only.

Biomarker	Serum +90d		Serum +180d	
	AUC	P value	AUC	P value
CCL4	.681*	**.019**	**.794**	**.004**
CXCL1	**.654**	**.047**	.664	.109
IL-6	.613	.146	**.727**	**.026**
PAI-1	**.696**	**.011**	**.804**	**.003**
CXCL9	.064	.179	**.724**	.029
CXCL11	**.701**	**.010**	.692	.060
sRAGE	**.675**	**.020**	**.769**	**.009**
PTX3	**.657**	**.043**	.626	.219
IL-10	.501	.987	**.787**	**.005**

*significant values are displayed in bold type with the corresponding p values.

In summary, in addition to biomarkers already described as diagnostic for cGVHD, we identified new serum proteins that are prognostic for cGVHD development in this observational study.

### Prognostic biomarkers in patients developing *de novo* cGVHD

**Figure 3:**
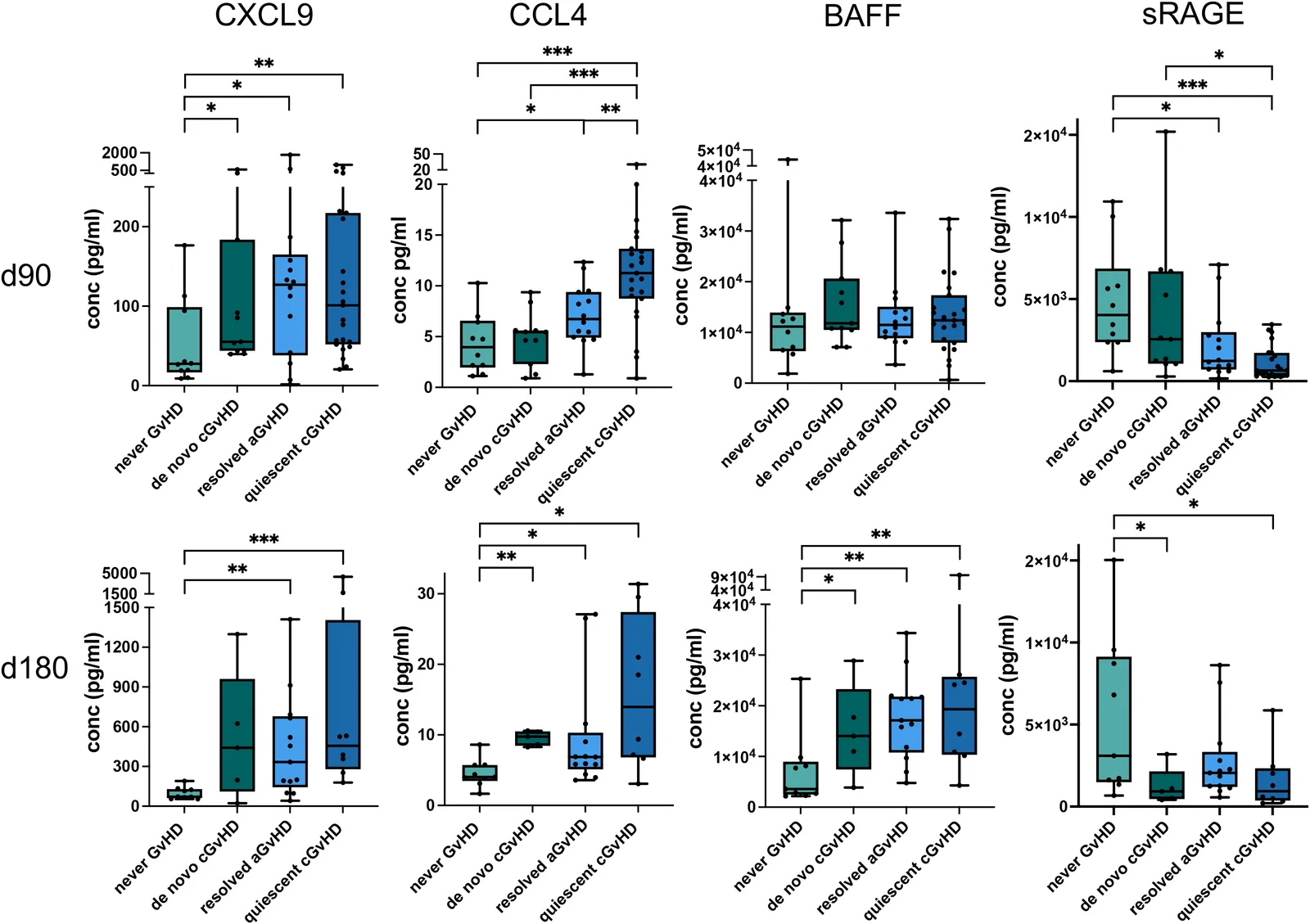
Cytokines and chemokines with prognostic increases or decreases in patients developing *de novo* cGVHD versus no GVHD. Serum concentrations of CXCL9, CCL4, BAFF and sRAGE +90 days (upper panel) and +180 days (lower panel) after allo-HSCT are shown as box plots. Serum levels for resolved aGVHD and quiescent cGVHD patients are displayed for comparison. p-values indicated as *p < 0.05, **p < 0.01, ***p < 0.001 by the Mann-Whitney U test.

At both time points after allo-HSCT, we intended to find prognostic biomarkers for subsequent *de novo* cGVHD or quiescent cGVHD. Four proteins showed potential to predict the development of *de novo* cGVHD within one year, compared to patients who never developed any form of GVHD. CXCL9 serum levels of the *de novo* cGVHD cohort were significantly increased compared to the never GVHD cohort at time point +90d thus predicting the subsequent cGVHD onset (**Figure 3**, p < 0.05). ROC analyses confirmed this result (**Table 3**). Interestingly, CXCL9 serum concentrations did not differ significantly between patients with resolved aGVHD and those with quiescent GVHD at either day +90 or day +180 (**Figure 3**). Patients who never developed any GVHD consistently had the lowest serum levels of CXCL9 at both time points (**Figure 3**). Also elevated CCL4 serum levels at day +180 were found to be prognostic for *de novo* GVHD (p < 0.01, **Figure 3**) and ROC analyses proved to be significant with an AUC of 0.978 (**Table 3**). A major focus of our study was the role of B–cell–modulating cytokines as prognostic biomarkers for subsequent cGVHD. B cell-activating factor (BAFF) showed prognostic potential for predicting the development of *de novo* cGVHD. At day +180, levels of BAFF were significantly higher in these patients compared to the patients who never developed GVHD (**Figure 3**, p < 0.05). ROC analyses proved to be significant with an AUC of 0.844 (**Table 3**). We hypothesized that anti-inflammatory cytokines contribute to the modulation of cGVHD development. Indeed, the soluble receptor for advanced glycation end products (sRAGE), an anti-inflammatory cytokine, exhibited a significant reduction in serum levels at day +180 in the *de novo* cGVHD cohort compared to the never GVHD group (**Figure 3**, p < 0.05). Also, ROC analyses were significant, with an AUC of 0.844 (**Table 3**).

**Table 3:** ROC analysis of the prognostic serum biomarkers and the occurrence of *de novo* cGVHD versus no GVHD.

Biomarker	Serum +90d		Serum +180d	
	AUC	P value	AUC	P value
CXCL9	.782*	**.029**	.800	.072
CCL4	.559	.647	**.978**	**.004**
BAFF	.664	.205	**.844**	**.039**
sRAGE	.618	.360	**.844**	**.039**

*significant values are displayed in bold type with the corresponding p values.

### Prognostic biomarkers in patients developing quiescent cGVHD from previous aGVHD

**Figure 4:**
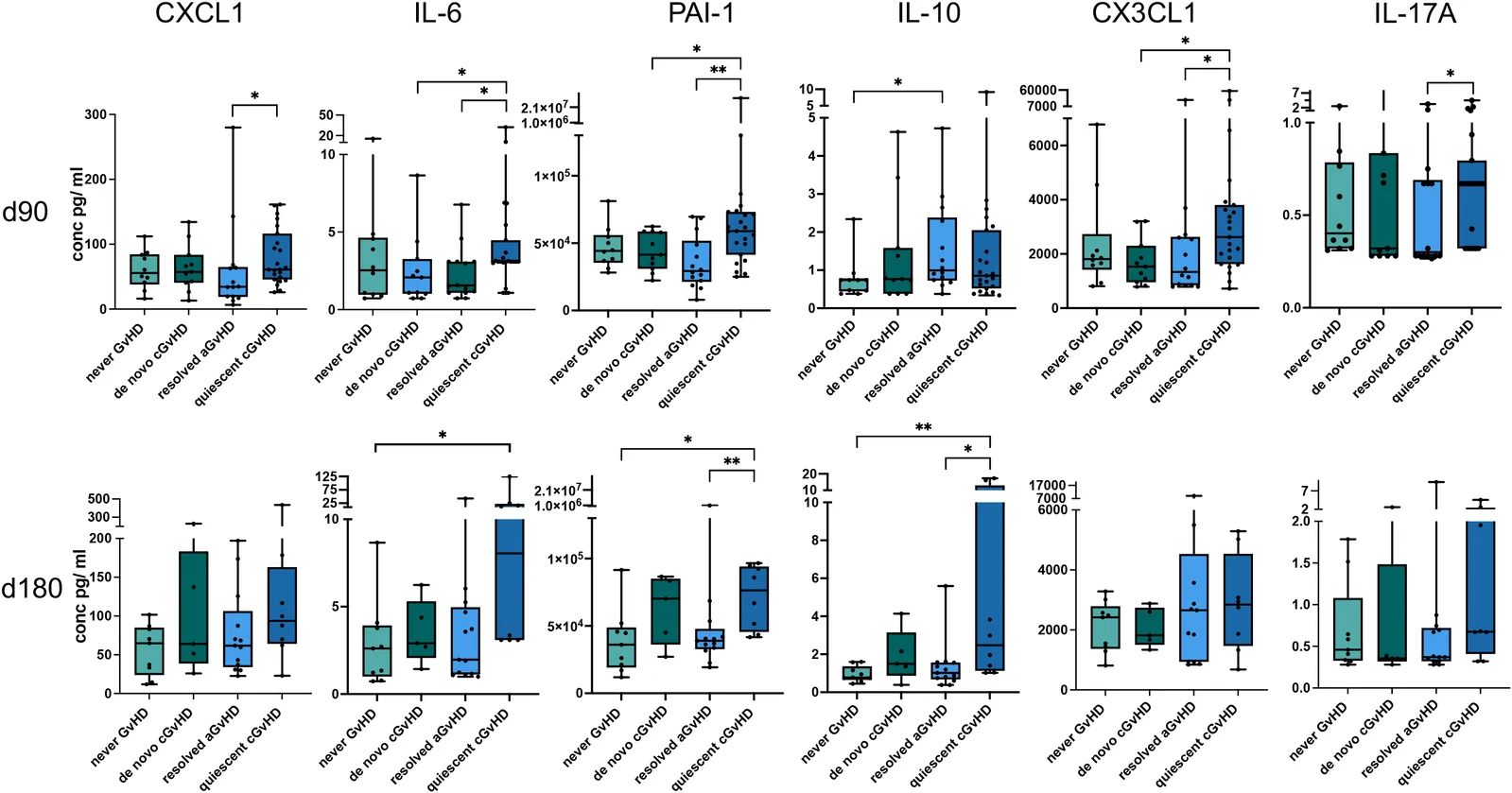
Cytokines and chemokines with prognostic increases or decreases in patients developing quiescent cGVHD versus patients with resolved aGVHD. Serum concentrations of CXCL1, IL-6, PAI-1, IL-10, CX3CL1 and IL-17A +90 days (upper panel) and +180 days (lower panel) after allo-HSCT are shown as box plots. Serum levels for never GVHD and *de novo* cGVHD patients are displayed for comparison. p-values indicated as *p < 0.05, **p < 0.01 by the Mann-Whitney U test.

Given the clinical impact of cGVHD, biomarkers capable of predicting its development after resolution of aGVHD are highly valuable. Thus, we compared serum markers for patients that did not develop cGVHD after a resolved aGVHD versus those that developed cGVHD within 1 year after HSCT. Seven proteins were significantly different between the two groups for either time point of analysis. As shown in **Figure 3**, CCL4 has also prognostic value for the development of quiescent cGVHD (p < 0.01) at the early time point, also reflected by ROC analysis (**Table 4**). Furthermore, CXCL1 showed significantly elevated levels at the +90d time point (p < 0.05, **Figure 4** and **Table 4**). Interestingly, IL-6 – an inflammatory cytokine – at +90d and the IL-6–induced plasminogen activator inhibitor-1 (PAI-1) at +90d and +180d were both markedly elevated in patients who later develop cGVHD following resolution of aGVHD (**Figure 4** and **Table 4**). IL-10, although generally considered anti-inflammatory, was also significantly elevated at the later time point (p < 0.05, **Figure 4** and **Table 4**). We further found a significant elevation of fractalkine/CX3CL1 in patients with quiescent cGVHD versus patients who resolved aGVHD without cGVHD development for the +90d time point (p < 0.05, **Figure 4** and **Table 4**). Likewise, a significant higher level of IL-17A was found in patients with quiescent cGVHD versus resolved aGVHD at day +90 (p < 0.05, **Figure 4** and **Table 4**). Interestingly, two well-established diagnostic biomarkers for cGVHD namely CXCL9 and BAFF showed no prognostic value for the development of cGVHD following preceding aGVHD in our study (**Figure 4**) (17).

**Table 4:** ROC analysis of the prognostic serum biomarkers and the occurrence of quiescent cGVHD versus aGVHD only.

Biomarker	Serum +90d		Serum +180d	
	AUC	P value	AUC	P value
CCL4	**.770**	**.006**	.702	.128
CXCL1	**.724**	**.024**	.683	.169
IL-6	**.728**	**.021**	.740	.070
PAI-1	**.809**	**.002**	**.846**	**.009**
IL-10	.598	.324	**.817**	**.017**
CX3CL1	**.714**	**.031**	.539	.828
IL-17A	**.696**	**.048**	.654	.247

*significant values are displayed in bold type with the corresponding p values.

## Discussion

To date, the exploration of prognostic biomarkers in cGVHD has been limited by the disease’s heterogeneity. In particular, the influence of ongoing aGVHD and the impact of corticosteroid immunosuppression on cytokine levels have complicated the search for reproducible prognostic markers in previous studies. Moreover, analyzing all cGVHD patients as a single group in terms of disease onset may have prevented the discovery of validated biomarkers that might be specific for a subcohort of cGVHD which has been recently described in children (29). This exploratory study lays the groundwork for a comprehensive biomarker analysis that should include a larger, multicenter cohort and independent validation.

One of the strengths of our study is the establishment of a clear framework to exclude cytokine alterations solely related to aGVHD by omitting all patients with ongoing aGVHD at the time of sample collection. In addition, we minimized the confounding effects of corticosteroid therapy by including only patients receiving ≤0.5 mg/kg body weight/day prednisone equivalent at sampling, as glucocorticoids can substantially influence serum protein expression (18, 24). Previous studies have primarily compared patients who developed cGVHD with those who did not, without accounting for the potential impact of different subcohorts on biomarker levels (22). Moreover, studies investigating risk factors for cGVHD have already recognized the need to distinguish between the two clinical types of cGVHD (30, 31). In our analysis, we therefore added a separate analysis distinguishing patients who developed *de novo* cGVHD from those who developed quiescent cGVHD and compared each group with matched controls - that is, *de novo* cGVHD with patients who never developed GVHD, and quiescent cGVHD with patients who had resolved acute GVHD, reflecting the clinical situation.

Acute GVHD is a well-established risk factor for the subsequent development of cGVHD (32, 33). It has been proposed that tissue damage caused by acute inflammation or its treatment promotes the onset of quiescent cGVHD (34, 35). These assumptions, however, do not apply to *de novo* cGVHD, except in cases where it follows a very mild untreated episode of aGVHD. In such instances, the pathogenesis – and consequently the contributing biomarkers – of *de novo* and quiescent cGVHD would be expected to overlap. In contrast, our findings provide evidence against this notion. We identified distinct prognostic biomarker panels predicting *de novo* versus quiescent cGVHD, suggesting that these two clinical entities may arise through partially distinct pathogenic mechanisms. These insights may help elucidate the biological underpinnings of cGVHD development.

Whereas CXCL9 was previously described as a diagnostic biomarker for cGVHD (17, 36), our data now identify elevated CXCL9 levels as a prognostic biomarker for *de novo* cGVHD. CXCL9 is strongly induced by IFN-γ (37). Binding to its receptor CXCR3 promotes the recruitment of activated lymphocytes to the inflammatory tissue (38, 39). In our cohort, CXCL9 serum levels were significantly increased 90 days after allo-HSCT in patients who later developed *de novo* cGVHD compared with their matched controls who never experienced GVHD. These findings suggest that IFN-γ–driven processes may contribute to the early pathogenesis of *de novo* cGVHD. In contrast, CXCL9 did not emerge as a prognostic marker for quiescent cGVHD, likely because CXCL9 serum levels were already elevated in patients who had resolved aGVHD, irrespective of subsequent cGVHD development.

The BAFF system is a key regulator of B-cell survival, maturation, and activation. At day +90 after HSCT, serum BAFF levels did not differ between patient cohorts. This likely reflects a general early post-transplant increase in B-cell–modulating cytokines, supporting reconstitution of the B-cell compartment after myeloablative conditioning. Echoing previous observations in patients with active cGVHD (18), elevated BAFF levels six months after transplantation were high in patients with the subsequent development of cGVHD but also found elevated in patients with aGVHD only. Importantly, our data now extend the prognostic association specifically to *de novo* cGVHD.

A novel finding of this study is the identification of the soluble form of the receptor for advanced glycation end products (sRAGE) as a potential biomarker for cGVHD. Advanced glycation end products (AGEs) constitute a heterogeneous group of molecules that interact with their membrane-bound receptor RAGE, triggering oxidative stress, pro-inflammatory cytokine production, and tissue inflammation (40). The soluble receptor sRAGE acts as a decoy by binding circulating AGEs and thereby inhibiting AGE–RAGE signaling and its downstream inflammatory effects (41). In our cohort, sRAGE emerged as a potential prognostic biomarker for *de novo* cGVHD, with significantly lower serum levels compared to patients who never developed GVHD. In contrast, sRAGE levels in patients who later developed quiescent cGVHD did not differ from those in their matched controls with resolved aGVHD.

CCL4 is a potent chemoattractant for monocytes and T cells that exerts its effects through binding to its receptor CCR5 (42). While CCR5 blockade has been shown to ameliorate visceral GVHD (43), CCL4 itself has not yet been described as a biomarker for any form of GVHD (42). In our cohort, CCL4 serum levels were elevated in patients with prior aGVHD and were even higher in those who subsequently developed quiescent cGVHD. Notably, CCL4 also demonstrated predictive value for *de novo* cGVHD, as shown here for the first time. Together, these findings identify CCL4 as a promising new candidate for a prognostic biomarker for both forms of cGVHD.

For the first time, we describe interleukin-6 (IL-6) as a promising prognostic biomarker for quiescent cGVHD since this patient cohort had the highest levels of IL-6. One explanation is that subclinical inflammatory activity persisting after aGVHD, despite clinical resolution, is captured by elevated IL-6 levels. This suggests that IL-6 may serve as a promising prognostic biomarker for identifying patients at risk of quiescent cGVHD early after aGVHD.

Th17 cells and their key effector cytokine IL-17A are known mediators in the pathophysiology of cGVHD, promoting disease progression toward sclerotic manifestations (27, 44, 45). In this study, we report for the first time that elevated IL-17A serum levels precede the development of quiescent cGVHD.

High PAI-1 levels are indicative of endothelial damage and have been linked to various vascular disorders (46–48). Nürnberger et al. reported elevated PAI-1 concentrations in several post-transplant complications, including GVHD (49). In our study, PAI-1 emerged as the most consistent prognostic biomarker for quiescent cGVHD, with significantly higher levels at both time points compared with patients who experienced but resolved aGVHD. Given that aGVHD causes substantial endothelial and tissue injury (27), we hypothesize that patients who do not progress to cGVHD can fully repair this damage, whereas those who develop quiescent cGVHD may fail to achieve complete endothelial recovery, leading to chronic disease (27).

The soluble chemokine CX3CL1, previously linked to tissue damage after conditioning in aGVHD showed elevated serum levels in patients who developed quiescent cGVHD compared to those with resolved aGVHD (27, 50). CX3CL1 levels were also higher in quiescent than in *de novo* cGVHD, supporting a mechanism driven by prior tissue injury.

Another pro-inflammatory chemokine, CXCL1, is a potent chemoattractant for neutrophils during inflammation (51). We find a potential in CXCL1 to serve as a prognostic biomarker for the development of quiescent cGVHD.

Interleukin-10 (IL-10) is a key anti-inflammatory cytokine. Ninety days after transplantation, elevated IL-10 levels were observed only in patients with resolved aGVHD, likely reflecting residual anti-inflammatory responses. At day +180, IL-10 was prognostically increased in patients who later developed quiescent cGVHD despite high inflammatory activity. Overall, IL-10 serum levels remained low and warrant further investigation.

Although our study identified several potential biomarkers with in part different performance depending on the type of onset of cGVHD, certain limitations should be acknowledged. Biomarker levels were not consistent across the two sampling time points, underscoring the need for longitudinal studies with more frequent sampling. The cohort size was limited, and larger patient populations will be required to replicate the findings. In addition, a larger patient population may reveal correlations between biomarker levels and cGVHD severity. In our cohort, we could not find such correlations (data not shown). We have not applied multivariate analysis as sample sizes were limited and therefore the identified biomarkers may not have independent prognostic information. A time-to-event framework would assess subsequent cGVHD risk and should be applied in future studies with larger patient numbers. Finally, this study was not designed to assess associations between biomarker levels and specific organ manifestations, which may influence serum protein profiles.

In conclusion, our study addresses the challenges of identifying prognostic biomarkers for cGVHD by applying a rigorous framework to minimize confounding factors. We identified several promising biomarkers for *de novo* cGVHD – particularly CXCL9, BAFF, and sRAGE – with strong predictive potential. In contrast, IL-6, IL-17A, PAI-1, CX3CL1, CXCL1, and IL-10 emerged as notable prognostic indicators for quiescent cGVHD, reflecting distinct underlying inflammatory processes. CCL4 was identified as a promising biomarker common to both subtypes. Together, these findings advance efforts toward more personalized prediction and management of cGVHD.

## Data Availability

The original contributions presented in the study are included in the article/**Supplementary Material**. Further inquiries can be directed to the corresponding author.
