# Correction: *De novo* and quiescent cGVHD are distinguishable in a prognostic biomarker panel

**DOI:** 10.3389/fimmu.2026.2025620

**Published:** 2026-10-20

**Authors:** Lara Vollmer, Katharina M. Habenicht, Andrea Schneider, Matthias Fante, Andreas Mackensen, Julia Winkler, Daniel Wolff, Thomas H. Winkler

**Affiliations:** 1 Department of Internal Medicine 5 – Hematology and Oncology, University Hospital Erlangen, Erlangen, Germany; 2 Department of Biology, Division of Genetics, Nikolaus-Fiebiger-Center for Molecular Medicine, Friedrich-Alexander-University Erlangen-Nuremberg, Erlangen, Germany; 3 Department of Internal Medicine III, University Hospital Regensburg, Regensburg, Germany

**Keywords:** acute GVHD, biomarker, chronic GVHD, disease onset, hematopoietic stem cell transplantation

In the published article, in the **Acknowledgements**, the following text was added to the previous version: The present work was performed in (partial) fulfilment of the requirements for obtaining the degree ‘Dr. med.’ for LV.

The original version of this article has been updated.

