# Supplementary material for: De novo and quiescent cGVHD are distinguishable in a prognostic biomarker panel
Source: Front Immunol. 2026 Feb 20;17:1760111. doi: 10.3389/fimmu.2026.1760111 (PMC12962918; doi:10.3389/fimmu.2026.1760111)
Supplement: Supplementary file 1 [file Table1.docx]

**Suppl. Table 1:** Median concentration and range (minimum – maximum) in pg/ml of all additionally analyzed biomarkers

|  |  |  |  |  |
| --- | --- | --- | --- | --- |
| Biomarker | **never GvHD** | **de novo cGvHD** | **resolved aGvHD** | **quiescent cGvHD** |
| APRIL d+90  d+180 | 4559 (2137–11428)  4337 (1008 – 9159) | 5163 (1886-28592)  10467 (2990-16137) | 3101 (51 –17234)  6143 (2031–66595) | 7014 (136 –14327)  8235 (1978-20601) |
| CCL2 d+90  d+180 | 290 (155–676)  225 (106–767) | 442 (170–841)  537 (179–692) | 416 (204–884)  410 (158–869) | 467 (169–1095)  617 (90–8974) |
| CCL11 d+90  d+180 | 50 (37–343)  50 (28–169) | 97 (35–334)  81 (70–232) | 150 (60–468)  111 (59–436) | 177 (53–848)  111 (37–193) |
| CCL17 d+90  d+180 | 211 (39–983)  339 (4–880) | 255 (12–1509)  415 (21–1149) | 163 (6–3504)  434 (29–3794) | 231 (8–1192)  195 (66–2792) |
| CXCL8 d+90  d+180 | 92 (68–631)  95 (48–371) | 68 (36–1043)  144 (68–790) | 68 (17–221)  68 (8–831) | 30 (9–1687)  67 (9–6204) |
| CXCL10 d+90  d+180 | 37 (15–163)  64 (34–181) | 48 (28–177)  204 (23–404) | 47 (10–809)  136 (36–690) | 45 (14–515)  438 (51–989) |
| CXCL12 d+90  d+180 | 363 (173–1182)  435 (112–6253) | 460 (143–837)  654 (105–938) | 329 (67–1217)  364 (128–1731) | 486 (142–2167)  476 (173–1123) |
| ENA-78 d+90  d+180 | 68 (11–264)  139 (1.4–282) | 104 (8–260)  157 (22–403) | 52 (1.0–254)  87 (5–3299 | 42 (12–313)  81 (19–280) |
| IL-2Ra d+90  d+180 | 20634 (1639–69855)  28016 (2252–53337) | 23922 (9242–42242)  35804 (12079-77854) | 20241 (1928–54289)  34091 (2367–102117) | 21733 (2400–67728)  32917 (8201–164022) |
| IL-4 d+90  Id+180 | 7 (1.5–52)  4 (0.9–37) | 1.8 (1.0–14)  5 (0.9–17) | 5 (0.9–19)  2 (1.2–24) | 5 (0.3–262)  5 (0.5–16) |
| IL-12p70 d+90  d+180 | 1.4 (0.6–7)  1.4 (0.6–21) | 1.2 (0.6–5)  1.3 (0.6–2) | 0.6 (0.6–4)  0.9 (0.6–4) | 0.7 (0.6–64)  1.3 (0.6–20) |
| sCD40L d+90  d+180 | 12708 (3485-28308)  13651 (1060–26667) | 15797 (3347–27706)  16047 (13543–26560) | 9374 (610–18796)  13387 (2132–26483) | 10926 (810–31151)  12052 (1888–22703) |
| sCD130 d+90  d+180 | 136569 (48229–312976)  129059 (48149–192947) | 105442 (64959–383022)  157888 (78275–308554) | 162244 (79263–434800)  121551 (56781–436905) | 209409 (105411–708473)  124897 (70338–256216) |
| sST2 d+90  d+180 | 507 (262–1402)  402 (160–864) | 426 (236–1872)  359 (238–1272) | 617 (193–6446)  488 (132–5976) | 1725 (253–23665)  2196 (204–8261) |
| sTNF-RI d+90  d+180 | 6229 (1841–7967)  3627 (2001–19536) | 3992 (1943–6743)  5562 (2513–7598) | 3690 (1520–9302)  3443 (1446–11075) | 4099 (1574–168768)  5214 (1546–9336) |
| sTNF-RII d+90  d+180 | 342 (127–670)  321 (127–1008) | 310 (195–1156)  28 (107–1566) | 375 (133–929)  459 (132–1582) | 318 (51–6772)  403 (57–1516) |
| sTREM-1 d+90  d+180 | 40 (21–130)  41 (24–58) | 25 (16–47)  27 (23–114) | 25 (14–830)  45 (14–704) | 43 (12–2638)  53 (9–132) |
| TNF-a d+90  d+180 | 12 (2–123)  9 (1.7–24) | 3 (1.1–38)  4 (2–37) | 4 (1.1–48)  4 (1.8–51) | 6 (0.3–325)  10 (0.5–29) |
